# GATv2EPI: Predicting Enhancer–Promoter Interactions with a Dynamic Graph Attention Network

**DOI:** 10.3390/genes15121511

**Published:** 2024-11-25

**Authors:** Tianjiao Zhang, Xingjie Zhao, Hao Sun, Bo Gao, Xiaoqi Liu

**Affiliations:** 1 College of Computer and Control Engineering, Northeast Forestry University, Harbin 150040, China; 2022122521@nefu.edu.cn (X.Z.); haosun@nefu.edu.cn (H.S.); 2 Department of Radiology, The Second Affiliated Hospital of Harbin Medical University, Harbin 150081, China; 602192@hrbmu.edu.cn; 3 Department of Orthopedic Surgery, The Second Affiliated Hospital of Harbin Medical University, Harbin 150081, China

**Keywords:** enhancer–promoter interaction, graph attention networks, epigenetics, gene regulation

## Abstract

Background: The enhancer–promoter interaction (EPI) is a critical component of gene regulatory networks, playing a significant role in understanding the complexity of gene expression. Traditional EPI prediction methods focus on one-to-one interactions, neglecting more complex one-to-many and many-to-many patterns. To address this gap, we utilize graph neural networks to comprehensively explore all interaction patterns between enhancers and promoters, capturing complex regulatory relationships for more accurate predictions. Methods: In this study, we introduce a novel EPI prediction framework, GATv2EPI, based on dynamic graph attention neural networks. GATv2EPI leverages epigenetic information from enhancers, promoters, and their surrounding regions and organizes interactions into a network to comprehensively explore complex EPI regulatory patterns, including one-to-one, one-to-many, and many-to-many relationships. To avoid overfitting and ensure diverse data representation, we implemented a connectivity-based sampling method for dataset partitioning, which constructs graphs for each chromosome and assigns entire connected subgraphs to training or test sets, thereby preventing information leakage and ensuring comprehensive chromosomal representation. Results: In experiments conducted on four cell lines—NHEK, IMR90, HMEC, and K562—GATv2EPI demonstrated superior EPI recognition accuracy compared to existing similar methods, with a training time improvement of 95.29% over TransEPI. Conclusions: GATv2EPI enhances EPI prediction accuracy by capturing complex topological structure information from gene regulatory networks through graph neural networks. Additionally, our results emphasize the importance of epigenetic features surrounding enhancers and promoters in EPI prediction.

## 1. Introduction

The occurrence of diseases is often related to abnormal gene expression, which can be influenced by various factors including genetic variants, interactions with other genes, the regulatory roles of transcription factors, and epigenetic modifications such as DNA methylation and histone acetylation [1,2]. Enhancers and promoters are cis-regulatory elements involved in gene expression [3,4], both being sequences of DNA. Enhancers play a promoting role in gene expression by influencing the expression levels of target genes, typically located tens of kilobases (kb) away from these genes [5,6,7]. Promoters act as the “switch” for gene expression, determining the initiation site for transcription and usually located just 1–2 kb from the target gene [3,8]. Enhancers exert their function by interacting with promoters, thereby increasing gene expression [3,9]. Although enhancers and their target promoters or genes are often distantly situated, their interaction (enhancer–promoter interaction, EPI) generally requires physical proximity or even contact [10,11]. The regulatory relationship between enhancers and target promoters is diverse and can typically be categorized into three modes: “many-to-one”, “one-to-many” and “many-to-many” [12]. Furthermore, gene expression exhibits tissue specificity, as different tissues possess distinct functions and structures, which is rooted in the tissue-specific regulation of gene expression. Consequently, the interactions between enhancers and promoters in different tissues also display significant tissue specificity [13,14].

From a methodological perspective, EPI prediction can be broadly classified into statistical methods, machine learning methods, and deep learning approaches. Traditional statistical methods often use distance, correlation, expression quantitative trait loci (eQTL), and chromatin interaction data, relying on simple mathematical models and prior assumptions [15,16,17]. Correlation-based methods are influenced by feature selection and the correlation algorithms used, and are sensitive to outliers, such as GeneHancer [18]. CT-FOCS [19] predicts EPIs that are active in only a few cell types using a linear mixed-effects model (LMM). EpiTensor [20] is a tensor decomposition-based model designed to infer three-dimensional genomic relationships from one-dimensional epigenomic data. These methods are advantageous due to their strong interpretability, low computational resource demands, and suitability for small datasets or limited-resource settings. However, they are limited by their reliance on simplified assumptions (like linear relationships and normal distributions), making it difficult to capture complex nonlinear features, and they generally perform worse than deep learning on large datasets.

Early machine learning approaches for EPI prediction, such as TargetFinder [21] and kmer-SVM [22], predicted using genomic signals and DNA sequence k-mer frequencies, but faced challenges in feature acquisition and generalization. 3DPredictor [23] defined EP interactions using quantified 3D interaction frequencies, providing a different perspective from traditional anchor-based methods. EP2vec [24] embedded DNA sequences using doc2vec and combined this with gradient boosting regression trees (GBRTs) for prediction, automatically learning features and capturing long-range dependencies. However, performance heavily depended on the choice of k values and the number of features. Compared to these methods, deep learning approaches can automatically extract complex nonlinear features without relying on feature selection.

Deep learning methods such as EPIANN [25], SPEID [26], SIMCNN [27], EPI-DLMH [28], ChINN [29], and EPI-trans [30] employ convolutional neural networks (CNNs), recurrent neural networks (RNNs), or Transformers to extract DNA sequence features. While these methods excel at feature extraction, they still primarily rely on sequence information. Studies have shown that local epigenomic features can provide richer information than sequences alone [31,32]. For example, DeepTACT [33] combines sequence information with chromatin accessibility data. However, many methods still neglect genomic signals from adjacent regions. In contrast, TransEPI [34] improves prediction accuracy by leveraging extensive genomic signals, though this approach may introduce feature redundancy and elevate computational demands. Issues with these methods include (1) the random division of datasets, leading to the same enhancers or promoters appearing in both training and testing sets, potentially overestimating model performance, as seen in TargetFinder, EPIANN, SPEID, SIMCNN, and ChINN; (2) unbalanced datasets leading to models biased towards predicting the majority class, i.e., negative samples, which may overlook the identification of truly interacting enhancer–promoter pairs, as in TransEPI and DeepTACT or over-sampling to increase the number of positive samples, as in EPI-DLMH and EPI-trans; and (3) traditional methods often simplify enhancer–promoter interactions into one-to-one relationships, but overlook the potential complex network relationships, such as multiple enhancers regulating the same promoter (or vice versa), thus failing to fully reflect their potential interactions and regulatory mechanisms.

Addressing the limitations of traditional EPI prediction methods, such as the inadequate consideration of one-to-many and many-to-many interaction modes, the selection of feature sets, and the rational division of datasets, we propose a novel predictive framework, GATv2EPI. This framework innovatively constructs a graph structure that includes enhancers, promoters, and their interrelationships, and utilizes convolutional neural networks (CNNs) along with dynamic graph attention networks (GATv2) [35] to deeply explore the complex regulatory relationships between enhancers and promoters. This approach comprehensively considers all interaction modes within EPI, emphasizing their importance in the regulation of gene expression. In terms of feature selection, GATv2EPI integrates various epigenetic features from enhancers, promoters, and their flanking regions, achieving an effective balance between feature richness and computational complexity. For dataset partitioning, GATv2EPI adopts a strategy based on graph connectivity, constructing datasets by sampling connected subgraphs from each chromosome. This method effectively prevents information leakage between training and testing sets and ensures consistent data distribution across different datasets. Overall, GATv2EPI provides a robust, network-based framework for predicting interactions between enhancers and promoters, enhancing prediction accuracy by capturing complex interaction modes such as one-to-many and many-to-many. This model offers deeper insights into the regulatory mechanisms underlying enhancer–promoter interactions.

## 2. Materials and Methods

### 2.1. Datasets

Our study utilized the BENGI dataset [36] to identify interactions between enhancers and promoters. This dataset integrates enhancer–gene interaction data from multiple biological samples, including experimental results based on 3D chromatin interaction technologies such as ChIA-PET, Hi-C, and CHi-C, as well as genetic interaction data identified through eQTL and CRISPR/dCas9 perturbation experiments. These comprehensive data provide an extensive benchmark for evaluating target gene prediction methods. For our study, we selected enhancer–gene interaction data identified via Hi-C technology, covering four cell lines: HMEC, IMR90, NHEK, and K562. These technologies were chosen for their efficiency and accuracy in revealing points of physical contact between functional elements within the three-dimensional chromatin structure, thereby aiding in the precise localization of direct interactions between enhancers and genes.

#### 2.1.1. Data Preprocessing

For the dataset obtained directly from BENGI, several preprocessing operations were necessary. This process consisted of four steps:

1. Positive sample selection: the original BENGI dataset contains both positive enhancer–gene (E-G) pairs and negative E-G pairs. Positive samples represent experimentally identified direct interactions, while negative samples mainly arise from two sources: interactions between enhancers and non-gene regions detected during experiments, and a large number of additional negative samples generated based on positive samples. Due to the extreme imbalance between positive and negative samples, and the potential for negative samples to represent enhancers interacting with non-gene regions, we filtered out all experimentally validated direct interactions from the BENGI dataset to serve as positive samples;
2. Mapping enhancer–promoter pairs: using the GENCODE annotation (v19), we mapped the original enhancer–gene pairs to more specific enhancer–promoter pairs, with promoters defined as 1500 bp upstream to 500 bp downstream of the transcription start site (TSS). This step allows for a more precise focus on key regions of transcriptional regulation, enhancing our understanding of regulatory complexity;
3. Statistics and filtering of EP distances: we conducted a detailed statistical analysis of the distances between enhancers and promoters in the positive samples using Python’s pandas and numpy libraries. Our analysis revealed that the majority of effective interactions occur within the range of 42 kb to 500 kb. Based on this finding, we specifically selected samples within this distance range for further study, ensuring the specificity and efficiency of our research;
4. Generation of negative samples: to address the common issue of sample imbalance in previous methods, we searched for promoters that did not show interactions within the same distance range for each positive enhancer, randomly selecting EP pairs that matched the number of positive samples to serve as negative samples. This approach ensures a near 1:1 ratio of positive to negative samples. Not only does this method balance the dataset, but it also enhances the robustness of the model’s predictive capability.

Through this series of optimization steps, our dataset not only retains the rigor of experimental design and the scientific integrity of the data but also significantly improves the balance and accuracy of the predictive model. This balanced approach increases the model’s sensitivity to the minority class, namely the actual enhancer–promoter interactions. Ultimately, we obtained 9403 positive samples and 9840 negative samples across these four cell lines, as detailed in Table 1.

#### 2.1.2. Dataset Partitioning Based on Data Connectivity

We employed a novel method based on data connectivity to partition the dataset of enhancer–promoter interactions. First, we constructed a graph on each chromosome, treating each enhancer and promoter as nodes, with their interactions—whether positive or negative samples—represented by edges. During the dataset partitioning process, we implemented a unique strategy: each connected subgraph (i.e., a complete and interconnected portion) was assigned to the same dataset. The goal of this approach was to ensure that no edges or nodes were shared between the training and testing sets. This strategy significantly reduces the potential for information leakage between the training and testing datasets, as it prevents specific graph structural features learned during training from reappearing in the testing set.

Moreover, our method guarantees that each dataset is drawn from samples across different chromosomes, enhancing sample diversity. This contrasts sharply with traditional methods that employ random partitioning or assign all samples from a single chromosome to either the training or testing set, which can lead to biases in chromosomal specificity of expression. By balancing samples across chromosomes, our approach not only ensures broad coverage and representativeness of the samples but also enhances the reliability and generalizability of the test results.

The primary advantage of this partitioning strategy lies in its ability to minimize information leakage while maintaining sample representativeness and diversity. By ensuring independence between the training and testing sets, our model can more accurately assess its generalization capabilities on unseen data, providing more reliable support for predicting gene regulatory mechanisms. This method not only strengthens the scientific rigor of the research but also offers a feasible and effective new strategy for future studies in complex biological network analysis.

Using this approach, we partitioned the dataset for each cell line into training, validation, and testing sets in a 3:1:1 ratio, ensuring that the proportion of samples from each chromosome in the training, validation, and testing sets also followed the same 3:1:1 distribution.

#### 2.1.3. Epigenetic Data

To delve deeper into the interactions between enhancers and promoters, this study took into account the influence of multiple epigenetic markers. The selected markers include H3K27me3, H3K36me3, H3K4me1, H3K4me3, H3K9me3, CTCF, and DNase-seq data, all of which are known to be crucial factors closely related to chromatin accessibility, transcriptional regulation, and chromatin structure regulation. H3K4me1, H3K4me3, and H3K27ac are generally enriched in transcriptionally active regions, especially near promoters and enhancers, and are associated with positive transcriptional activity. H3K9me3 and H3K27me3 are usually considered epigenetic marks of transcriptional repression, enriched in silenced regions. Although H3K36me3 is typically associated with transcriptional elongation, it has shown a negative correlation with eRNA expression in certain contexts, potentially having an inhibitory effect on enhancer function. CTCF, a key regulatory factor in chromatin loop structures, frequently binds at sites between enhancers and promoters, helping to maintain the regulatory network of gene expression. Additionally, DNase-seq, by measuring DNase I hypersensitive sites, identifies open chromatin regions, providing critical information for understanding transcription factor binding and gene regulation.

All selected ChIP-seq and DNase-seq data were obtained from the ENCODE (Encyclopedia of DNA Elements) database, specifically the hg19 version. These data are provided in bigBed format, with selected peak files that have multiple replications (replicate 1 and 2) to ensure the reproducibility and reliability of the results. The downloaded bigBed files were first converted into bed format to facilitate subsequent analysis. The conversion process utilized the UCSC’s bigBedToBed tool, ensuring uniform data formatting and smooth progression of subsequent processing. Afterwards, peak signal intensities were extracted from the bed files, representing the enrichment levels of various epigenetic markers at specific genomic locations. This step is crucial for quantifying the presence and potential regulatory impact of these markers across different genomic regions.

### 2.2. GATv2EPI Model Framework

**Figure 1:**
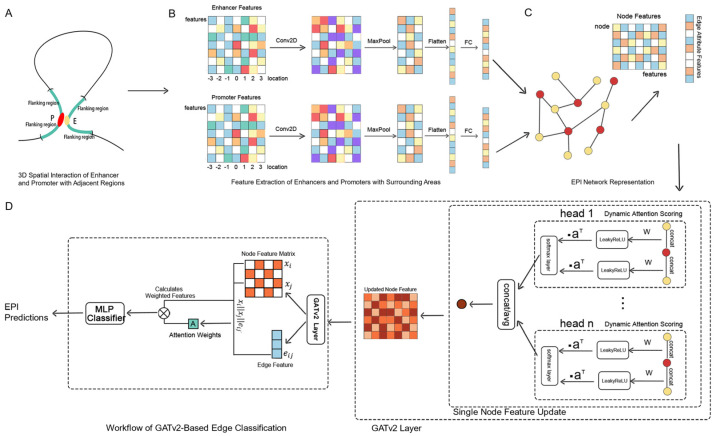
GATv2EPI model framework. (**A**) EPI feature region selection model diagram. Red and yellow represent enhancers and promoters, respectively, while the green section indicates the flanking regions of the enhancer–promoter pairs. (**B**) Feature extraction. Features are extracted from the enhancers, promoters, and their surrounding areas. The resultant feature vectors are used to construct the node features within the graph. (**C**) Construction of the EPI graph structure. The extracted features of enhancers and promoters are represented as nodes, with edges representing their spatial association relationships. (**D**) EPI prediction based on GATv2. GATv2 dynamically calculates attention coefficients and incorporates a multi-head attention mechanism to update node expressions. The features of the edges and adjacent nodes are then utilized to classify the enhancer–promoter (EP) pairs.

We propose a hybrid model that combines convolutional neural networks (CNNs) and graph neural networks (GNNs) to effectively extract complex features from genomic data. The model is divided into two components: the CNN processes the local features of each node, while the GNN captures the topological relationships and interactions between nodes within the graph structure. This model is capable of learning rich information from both the local features of nodes and the graph structure simultaneously, making it well-suited for analyzing the intricate interactions between enhancers and promoters within gene regulatory networks. The overall model framework is illustrated in Figure 1.

#### 2.2.1. Generation of the Feature Matrix

To investigate the interactions between enhancers and promoters, we focused on analyzing the genomic features surrounding these gene regulatory elements. We selected the enhancers, promoters, and their flanking regions of fixed lengths as the primary subjects for analysis. This selection is based on the hypothesis that genomic features near regulatory elements have a more significant impact on their regulatory activity than those in distal regions.

For precise processing and analysis of these genomic regions, we employed the pybedtools library, a widely used tool in bioinformatics analysis. During feature extraction, we applied masking techniques to exclude other potential regulatory elements within the analysis regions to avoid interference. Additionally, given the significant variation in the signal peaks of different genomic markers (e.g., H3K27me3, H3K36me3, H3K4me1, H3K4me3, H3K9me3, CTCF, and DNase), we set the promoter length to 2 kb as the size of the flanking windows, adopting a fine-grained window segmentation approach for feature extraction. Specifically, we extended 10 windows of 2 kb each to the left and right flanking regions of each enhancer or promoter, combined with the enhancer or promoter region itself, forming a total of 21 windows. The window size was chosen considering the variable lengths of enhancers and was defined based on promoter length. This design not only covers the regulatory element itself but also includes its surrounding genomic environment, enabling a more accurate capture of genomic feature variations near the regulatory elements.

For each window, we collected and analyzed the signals from the seven aforementioned genomic markers. To ensure the accuracy and consistency of the feature data, we applied normalization procedures to these data. This included calculating the mean and standard deviation of each feature and then standardizing them accordingly. Additionally, we employed min–max normalization to scale all feature values to the same numerical range, thereby reducing bias during model training. The final generated feature matrix is a 7 × 21 matrix, where each row represents the signal of a different genomic marker, and each column corresponds to a specific window position.

#### 2.2.2. Local Feature Extraction

The CNN component consists of two independent CNN modules (cnn_e and cnn_p), which are responsible for extracting local features from enhancer and promoter nodes, respectively. Each module uses separate feature channels, with each channel corresponding to a specific biological marker or genomic feature, ensuring that the network learns local patterns independently for each feature. This design allows the convolution operations to focus on multiple positions (windows) of a single feature. By setting the convolution kernel size to (1, kernel_size), the convolution only operates along the width dimension of the features, without crossing feature channels. This configuration helps to uncover biological signals in specific regions, identifying local areas that may play critical roles in biological functions.

The LeakyReLU activation function prevents neuron inactivity, while the MaxPool2d layer reduces the feature dimensions while preserving key information. The output is then flattened and passed through a fully connected layer to map it into a new feature space, providing integrated inputs for the GATv2 layer. Additionally, the node features extracted from the CNN are concatenated with the node’s structural features (e.g., node type and node degree), forming a complete set of graph node features. In summary, the CNN module focuses on feature extraction for individual nodes, without considering the relationships between nodes in the graph structure.

CNN:

(1) $$x_{conv}=LeakyReLu\left(W_{conv}*x+b\right),$$

(2) $$x_{pool}=MaxPool\left(x_{conv}\right),$$

(3) $$x_{cnnout}=W_{fc}.Flatten\left(x_{pool}\right)+b_{fc},$$

where $W_{conv}$ are the learnable convolution weight parameters, ∗ denotes the convolution operation, b is the bias term, and LeakyReLu is the activation function. $W_{fc}$ is the weight matrix of the fully connected layer, and $b_{fc}$ is the bias term.

#### 2.2.3. Constructing the Graph Structure

To integrate the genomic signal information of enhancers and promoters with the topological structure of the existing enhancer–promoter interaction (EPI) network, we transformed the traditional paired enhancer–promoter pairs into an enhancer–promoter interaction network graph. In constructing the graph structure of the EPI, all enhancers and promoters are treated as nodes, and additional features are defined for each node, such as node degree and one-hot encoded node type. This information helps capture the role and significance of each node within the regulatory network.

Additionally, we defined features for each edge in the graph, such as the actual distance between enhancers and promoters and the type of edge, which are crucial for understanding the nature of interactions between nodes. In the testing set, the edge type feature was designed to be masked, allowing us to evaluate the model’s generalization ability without directly using this information. Through this detailed data preparation and analysis process, we aim to gain deeper insights into the dynamic interactions between enhancers and promoters within gene regulatory networks, providing a more precise model for studying gene expression regulatory mechanisms.

For node types, we employed a one-hot encoding approach:

(4) $$x_{type}=\left\{\begin{array}{c} \left[0,1\right],if\;type\;is\;e\\ \left[1,0\right],if\;type\;is\;p \end{array}\right.,$$

We then concatenate the CNN output $x_{cnnout}$, node degree $x_{degree}$, and node type $x_{type}$:

(5) $$x=cancat\left(x_{cnnout},x_{degree},x_{type}\right),$$

#### 2.2.4. EPI Prediction Based on GATv2

To capture complex relationships within graph structures, we employed GATv2. While traditional GAT can assign varying attention scores to neighboring nodes, it suffers from a significant limitation: its static attention mechanism. Specifically, for any query node, the attention score rankings for neighboring nodes remain fixed and are not influenced by the query node’s characteristics. This restriction limits GAT’s effectiveness in handling intricate graph problems, particularly when it fails to differentiate between a query node and its various neighbors, leading to inadequate learning of node interrelations and overall model performance.

In contrast, GATv2 introduces a dynamic attention mechanism by adjusting the computation order of attention coefficients. This allows attention weights to depend not only on neighboring nodes but also to be dynamically adjusted based on the query node’s features. Consequently, the model can flexibly allocate attention to neighboring nodes according to specific contexts, enhancing its expressive capability. The dynamic attention mechanism of GATv2 is particularly effective in discerning subtle differences between enhancers and promoters, facilitating a nuanced approach to their interactions.

Additionally, we implemented a multi-head attention mechanism in GATv2, enabling independent computation of relationships between nodes and their neighbors while aggregating features from multiple subspaces. This approach enhances the diversity and comprehensiveness of node representations. After processing node features, we introduced edge features and utilized an Edge Attention Classifier for edge classification, successfully transforming the EPI prediction task into an edge classification challenge.

Specifically, the process of utilizing GATv2 for EPI prediction can be broken down into the following steps:

Node feature processing: concatenate the original feature vectors of each node i and its neighbor j, $x_{i},$ and $x_{j}$. If edge features are present, include the edge features $e_{ij}$:

(6) $$x=cancat\left(x_{i},x_{j},e_{ij}\right),$$

Dynamic attention computation: apply a learned weight matrix W to the concatenated features $x_{i,j}^{\prime }$ for linear transformation, followed by a LeakyReLU activation, and compute the inner product with an attention vector a to obtain the attention scores:

(7) $$e\left(x_{i},x_{j}\right)=a^{T}.\mathrm{LeakyReLU}\left(W.\;\;x_{i,j}^{\prime }\right),$$

Normalize scores across all neighbors j of node i using softmax:

(8) $$a_{ij}=softmax\left(e\left(x_{i},x_{j}\right)\right),$$

Multi-head attention mechanism: each head computes node features independently and aggregates them, enhancing representation diversity. The feature update for node iii in head h is computed as follows:

(9) $$x_{i}^{new}=\sigma \left(\sum_{j\in N_{i}}a_{ij}\;Wx_{i,j}^{\prime }\right),$$

where σ is a non-linear activation function. Aggregate features from each head:

(10) $$x_{i}^{\prime }=cancat\left(x_{i}^{1},x_{i}^{2},x_{i}^{3},\ldots ,x_{i}^{H}\right)\;\;\;or\;\;x_{i}^{\prime }=\frac{1}{H}\sum_{h=1}^{H}x_{i}^{h},$$

Edge classification handling: after updating node features, concatenate the transformed features of nodes i and j with edge features $e_{ij}$ for edge classification:

(11) $$e_{ij}^{\prime }=cancat\left(x_{i}^{\prime },x_{j}^{\prime },e_{ij}\right),$$

Pass the concatenated features through a linear layer followed by ReLU and softmax for normalization to compute edge attention weights, which update node features and produce final outputs through another linear layer:

(12) $$p=W_{clas}\left(\mathrm{ELU}(a_{e}.e_{ij}^{\prime })\right)+b_{clas},$$

where $a_{e}$ represents the attention weights for the features of each edge, which are obtained from the edge features processed through the attention mechanism. Finally, p represents the logits, or raw prediction scores, used for calculating loss.

This model design effectively addresses both local feature handling and complex interactions within graph structures, capturing the intricate dynamics of enhancer–promoter relationships.

### 2.3. Model Training and Evaluation

In our study, we implemented strategies to enhance model training effectiveness and result reliability.

Subgraph partitioning and parallel training: we partitioned subgraphs by chromosome and conducted parallel training. This approach leverages PyTorch Geometric’s design, facilitating parameter sharing across subgraphs, which conserves memory and improves training efficiency.

Loss function design: we utilized a combined loss function integrating binary cross-entropy loss and hinge loss:

(13) $$L\left(p,y\right)=w.BCE\left(p,y\right)+\left(1-w\right)Hinge\left(p,y\right),$$

The BCE is defined as follows:

(14) $$BCE\left(p,y\right)=-\frac{1}{E}\sum_{i=E}^{E}\left[y_{i}.\log\left(\sigma \left(p_{i}\right)\right)+\left(1-y_{i}\right)\log\left(1-\sigma \left(p_{i}\right)\right)\right],$$

Hinge loss is as follows:

(15) $$Hinge\left(p,y\right)=\frac{1}{E}\sum_{i=E}^{E}\max\left(0,1-y_{i}^{\prime }p_{i}\right),$$

where w is the weight for the binary cross-entropy loss, and $y_{i}^{\prime }$ is the transformed label. This combination enhances classification accuracy and robustness, especially near decision boundaries.

We evaluate our model using two metrics: the Area Under the Receiver Operating Characteristic Curve (AUC) and the Area Under the Precision–Recall Curve (AUPR). The ROC curve is constructed by plotting the True Positive Rate (*TPR*) against the False Positive Rate (*FPR*) at various threshold settings. *TPR*, also known as sensitivity or recall, measures the proportion of actual positive samples that are correctly identified by the model as positive. *FPR*, referred to as specificity, measures the proportion of actual negative samples that are correctly identified via the model as negative. Precision indicates the proportion of predicted positive samples that are actually positive.

(16) $$TPR=\frac{TP}{TP+FN},$$

(17) $$FPR=\frac{TP}{FP+TN},$$

(18) $$Precision=\frac{TP}{TP+FP},$$

where TP and TN represent the correctly predicted positive and negative samples, respectively, while FP and FN represent the incorrectly predicted positive and negative samples, respectively.

### 2.4. Data Visualization and Analysis Tools

For statistical analysis and result visualization in this study, we utilized several well-established Python libraries. These tools were carefully selected for their robust analytical capabilities and widespread acceptance in the scientific community, ensuring both the clarity of our data presentation and the reproducibility of our results. Specifically, Pandas, NumPy, and SciPy were employed for data processing, analysis, and statistical modeling; NetworkX was used to analyze and visualize the complex network relationships between enhancers and promoters; Matplotlib and Seaborn generated all visual representations; and the Time library, a standard tool for handling time and dates, was used to assess the model’s time performance.

## 3. Results

### 3.1. Assessment of EPI Prediction by GATv2EPI

**Figure 2:**
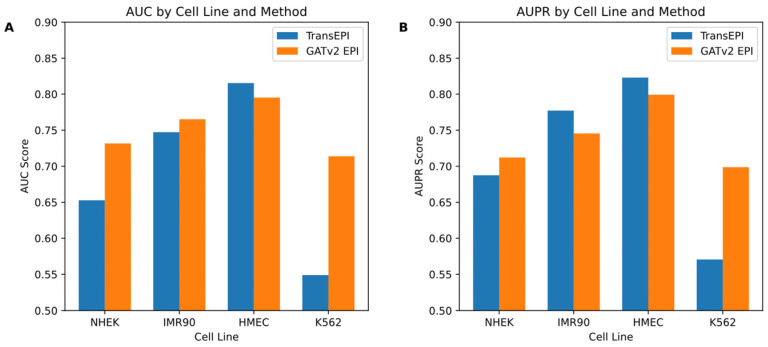
Comparative performance of TransEPI and GATv2 EPI across different cell lines. If there are multiple panels, they should be listed as follows: (**A**) the bar chart displays AUC scores for the TransEPI and GATv2 EPI methods across four cell lines: NHEK, IMR90, HMEC, and K562; (**B**) this panel shows AUPR scores for the same methods and cell lines.

In this study, we initially assessed the efficacy of the GATv2EPI model compared to the TransEPI model in predicting enhancer–promoter interactions (EPIs) across various cell lines (NHEK, IMR90, HMEC, and K562). As shown in Figure 2, the results for TransEPI were obtained from the average of five-fold cross-validation based on multiple experiments, whereas the results for GATv2EPI were derived from the final experimental outcome. By evaluating the models using two widely recognized metrics—the Area Under the Curve (AUC) and the Area Under the Precision–Recall Curve (AUPR)—we observed that GATv2EPI consistently outperformed TransEPI, especially showing significant advantages in the K562 cell line, which is commonly associated with leukemia. These observations suggest that GATv2EPI may offer higher sensitivity and accuracy in processing data related to disease-specific cells.

**Figure 3:**
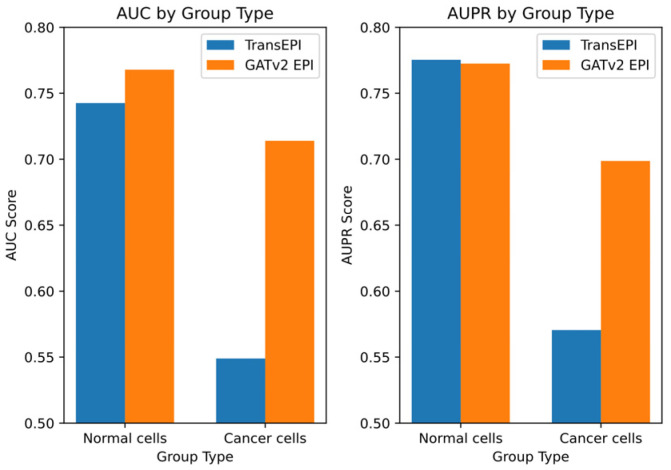
Comparative performance of TransEPI and GATv2EPI models in normal and cancer cells. The left panel shows the AUC scores, while the right panel displays the AUPR scores.

Building on these initial results, we designed a further experiment to explore the differential performance of GATv2EPI between normal cells and cancer cells. We categorized data from the HMEC, IMR90, and NHEK cell lines as ‘normal cells’ and data from the K562 cell line as ‘cancer cells’, establishing two distinct EPI prediction models. The dataset for the normal cells comprised 7053 positive samples and 7459 negative samples, while the dataset for the cancer cells contained 2350 positive and 2381 negative samples. This categorization enabled targeted predictive testing on both datasets, with the results depicted in Figure 3.

From these tests, GATv2EPI demonstrated superior performance over TransEPI in terms of AUC in both cell groups. Regarding AUPR, GATv2EPI’s performance equaled that of TransEPI in the normal cell group but was significantly better in the cancer cell group. Particularly for data related to diseases, the enhanced performance of GATv2EPI highlights its robust adaptability and potential in managing complex disease states. These findings not only confirm the effectiveness of GATv2EPI but also underscore its unique advantages in data processing under disease conditions.

In conclusion, although the performance differences between GATv2EPI and TransEPI were minimal in normal cells, the distinct superiority of GATv2EPI in cancer cells underscores its broader application prospects and potential value in disease data processing. Future research could further investigate the performance of GATv2EPI across a broader array of disease datasets, thereby confirming its suitability across various disease contexts.

### 3.2. Training Time of GATv2EPI Model

In this study, we significantly improved the training efficiency of the GATv2EPI model by partitioning subgraphs according to chromosomes and parallel training on different subgraphs while sharing model parameters. Experiments were conducted on a Linux server equipped with an Intel Xeon Gold 6326 CPU, two NVIDIA A100 GPUs (each with 80GB of VRAM), and 1.0 TB of memory. The deep learning framework used was PyTorch 1.10.1 + cu111, which supports CUDA 11.2 and driver version 460.106.00, with the code written in Python 3.9.17. Due to the shared nature of the server’s VRAM resources, the batch size for the TransEPI model was limited to 64. In contrast, the GATv2EPI model utilized a strategy of subgraph partitioning by chromosome, setting its geometric batch size to 1, allowing for more flexible VRAM usage and effectively preventing out-of-memory issues. Both models were trained for 200 epochs on the same dataset, which included 14,512 normal cell samples and 4731 K562 disease cell samples. As shown in Table 2, the training time for TransEPI on the K562 dataset was 396.57 s, whereas GATv2EPI required only 44.24 s. On the normal cell dataset, TransEPI took 1001.41 s to train, while GATv2EPI only needed 47.18 s. These data underscore the efficiency and speed advantages of GATv2EPI when handling large-scale and complex graph-structured data.

Several factors contribute to the faster training speed of our model. First, there are differences in the range and dimensionality of feature extraction. TransEPI extracts features from a 2.5 Mbp range, comprising 5000 bins, each containing eight dimensions of features, resulting in a large total feature count. In contrast, GATv2EPI extracts features from a 40 kb range, consisting of 21 bins with seven dimensions each. This significant reduction in feature count greatly diminishes computational complexity, thereby enhancing training efficiency. Secondly, the model architectures differ substantially. TransEPI combines CNN and Transformer structures, while GATv2EPI is based on CNN and graph attention networks (GATv2). The GATv2 model is designed with consideration for the sparsity of graph data, demonstrating greater efficiency and accuracy in handling sparse graph data compared to the large matrix operations required by Transformer models. Transformer models process sequence data using global self-attention mechanisms, which involve considerable computation and memory consumption; in contrast, GATv2 focuses local attention on neighboring nodes, significantly reducing computational complexity and optimizing resource usage. Furthermore, our training strategy of partitioning subgraphs by chromosome not only saves VRAM but also accelerates training speed. By dividing large-scale graphs into multiple subgraphs and training each independently, we can more efficiently utilize GPU resources. In comparison, TransEPI’s training time is prolonged due to its limited batch size imposed by VRAM constraints. Consequently, GATv2EPI exhibits superior performance in terms of computational time and resource consumption.

### 3.3. Key Signal Regions of Promoters and Enhancers

**Figure 4:**
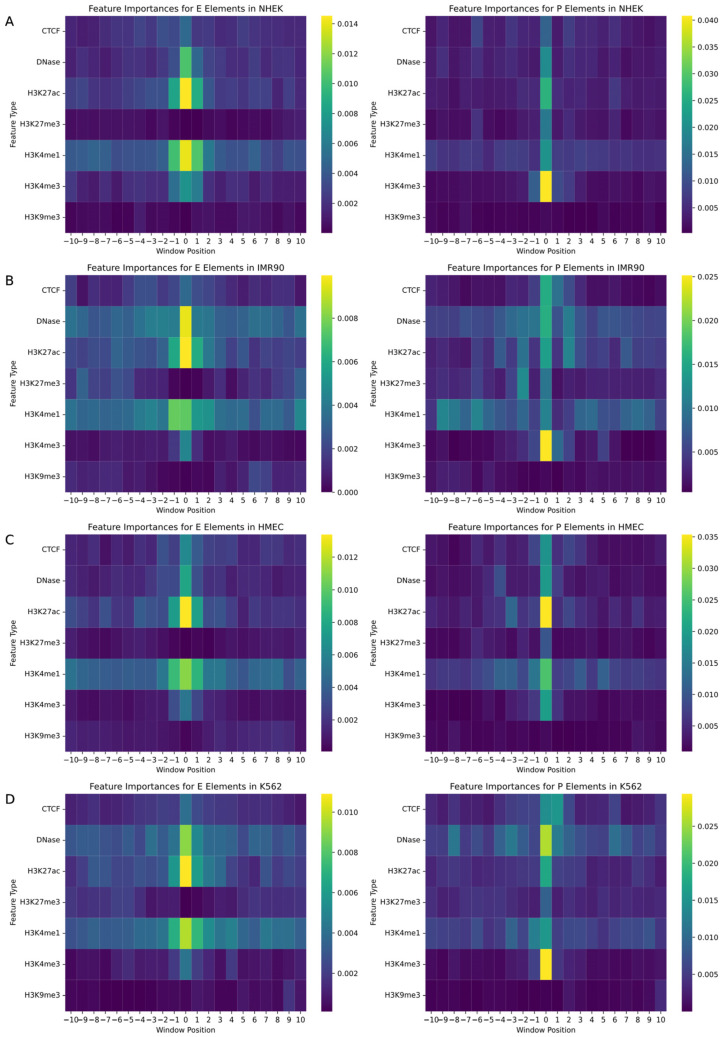
Heatmaps of feature importance for enhancers and promoters in gene regulatory networks. This figure illustrates the feature importance across various genomic positions relative to enhancers and promoters for four different cell types. Panels (**A**–**D**) represent the heatmaps for different cell types: (**A**) NHEK, (**B**) IMR90, (**C**) HMEC, and (**D**) K562. The heatmaps display the relative importance of specific genomic features, such as transcription factors and histone modifications, within a window ranging from −10 to +10 around the enhancer or promoter regions. The x-axis indicates the relative genomic position, while the y-axis shows different feature types. The color scale represents the magnitude of feature importance, where warmer colors indicate higher importance.

In the study of gene regulatory networks, accurately understanding the biological features and their specific locations within the genome is crucial for predicting interactions between enhancers and promoters. This research employed a random forest model to quantify feature importance, aiming to identify decisive biomarkers and their positional information in predicting enhancers and promoters. We assessed a variety of biological signals including CTCF, DNase, and various histone modifications such as H3K27ac, with each feature marked within a −10 to +10 window around the enhancer or promoter. The random forest model enabled the generation of two heatmaps for each cell line, mapping the importance of features for both promoters and enhancers, as illustrated in Figure 4.

The analysis revealed that biomarkers closely associated with the physical locations of enhancers and promoters had the most significant impact on EPI prediction. Particularly, signals located at the central position (window position 0) near enhancers and promoters exhibited the highest feature importance. Additionally, the importance of signals significantly decreased with increasing distance from enhancers or promoters, emphasizing the pivotal role of biomarkers near regulatory regions in predicting gene regulatory events. Histone modifications such as H3K4me3, H3K4me1, H3K27ac, and DNase I hypersensitive sites showed high feature importance across all cell types due to their role in identifying active gene regulatory regions, becoming key features for predicting interactions between enhancers and promoters. Moreover, specific biomarkers like CTCF binding sites were more important in promoter predictions in certain cell lines, while they might not be the most critical feature in enhancer predictions, reflecting functional differences in various regulatory contexts. Although some biomarkers are universally important across all cell lines, the specific degree of importance and the range of positions show significant variability among different cell types. For instance, in HMEC cells, the prominence of H3K27ac near enhancers may exceed that in other cell types, reflecting cell-type-specific gene regulatory mechanisms and expression patterns.

Through these detailed analyses, we not only gained a deeper understanding of the role of specific biomarkers in gene regulatory networks but also revealed the behavioral patterns of these features in different cellular environments. These findings have significant scientific and clinical implications for the precise regulation of gene expression, offering directions for future research and potentially guiding the development of gene therapy and precision medicine.

### 3.4. Analysis of Differences in Enhancer–Promoter Interactions Among Cell Lines

**Figure 5:**
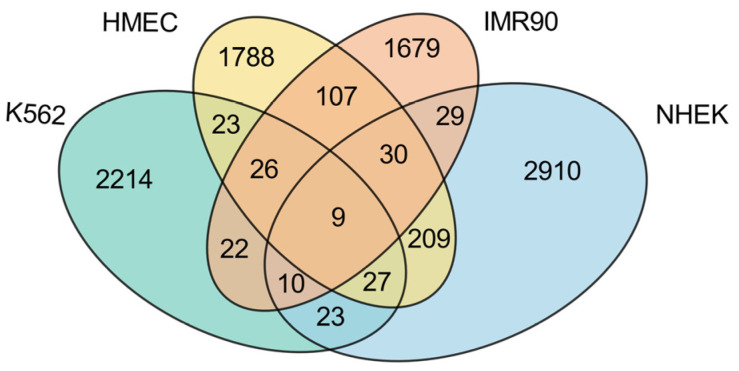
Intersection analysis of enhancer–promoter interactions across different cell lines. This Venn diagram displays the overlaps and distinctions in enhancer–promoter interactions among four cell lines: K562, HMEC, IMR90, and NHEK. The intersections highlight shared and unique regulatory connections.

This study conducts a detailed analysis of the intersection of enhancer–promoter interactions (EPIs) across four cell lines (K562, HMEC, IMR90, and NHEK), revealing both commonalities and specificities within the gene regulatory networks of these cell lines, as illustrated in Figure 5.

First, examining the proportion of cell line-specific EPIs, each cell line exhibits a high level of unique EPIs, with K562 showing the highest proportion at 92.99%, significantly exceeding those of the other normal cell lines. This indicates that the K562 cell line, being a cancer cell line, has a relatively unique regulatory network, likely to support its rapid growth and proliferation. In contrast, the HMEC cell line presents the lowest proportion of unique EPIs at 79.61%, which may reflect a higher degree of commonality in gene regulatory mechanisms with other normal cell lines. The presence of numerous unique EPIs suggests that each cell line possesses its own distinct regulatory network.

While most EPIs display high specificity across the different cell lines, there is also a notable number of shared EPIs. Among the normal cell lines, HMEC, IMR90, and NHEK share a considerable number of EPIs, with HMEC sharing 172 EPIs with IMR90 and 275 EPIs with NHEK, while IMR90 and NHEK share 78 EPIs. These data indicate that there are many similarities in gene regulation among normal cell lines, particularly highlighting HMEC’s significant role in sharing with others, potentially due to similarities in tissue type or physiological function. Conversely, K562 shares relatively fewer EPIs with the normal cell lines, specifically 85 with HMEC, 67 with IMR90, and 69 with NHEK. This suggests a marked difference in gene regulatory mechanisms between the cancer cell line and the normal cell lines. Notably, across all four cell lines, only nine EPIs are commonly shared, as detailed in Table 3. These shared EPIs may represent fundamental gene regulatory mechanisms that play core roles in all cells, likely closely related to the maintenance of essential biological functions.

In summary, these findings indicate that K562, as a cancer cell line, exhibits strong specificity, with a regulatory network significantly distinct from that of normal cell lines. However, among the normal cell lines, HMEC shows the highest number of shared EPIs, possibly due to its greater universality and adaptability within gene regulatory networks. This characteristic allows HMEC to display more commonalities in comparison with other normal cell lines. Conversely, the lower number of shared EPIs between IMR90 and NHEK further emphasizes that the specificities and commonalities among different cell lines are multi-layered, transcending a simple dichotomy between cancer and normal cell lines.

### 3.5. Analysis of EPI Network Structural Characteristics Across Different Cell Lines

This study aimed to systematically analyze the structural characteristics of enhancer–promoter interaction (EPI) networks in four cell lines (K562, HMEC, IMR90, and NHEK).

**Figure 6:**
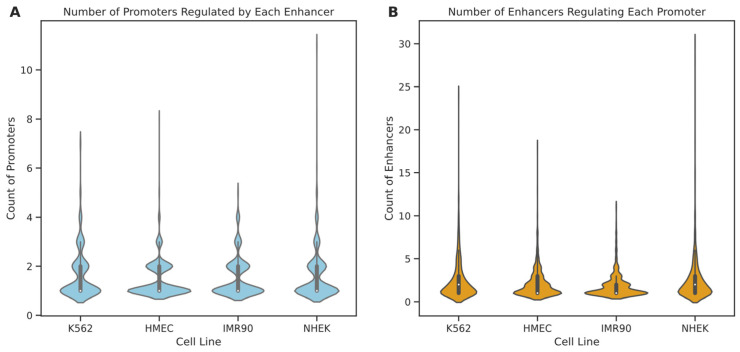
Distribution of node degrees for enhancers and promoters across different cell lines. This figure illustrates the distribution of node degrees within EPI networks for various cell lines, where the node degree represents the number of connections to other regulatory elements. Panel (**A**) shows the distribution of the number of promoters regulated by each enhancer, while panel (**B**) illustrates the distribution of the number of enhancers regulating each promoter. Violin plots are used to effectively convey the range and density of the data, revealing the complexity of regulatory interactions and highlighting the potential roles of enhancers and promoters in regulating gene expression within EPI networks.

Initially, we analyzed the degree distribution of enhancer and promoter nodes within these cell lines (see Figure 6). The results indicate that in all four cell lines, 75% of enhancers regulate only one to two promoters, suggesting that most enhancers tend to have specific interactions with a few promoters. A minority of enhancers interact with multiple promoters, with enhancers in IMR90, K562, HMEC, and NHEK engaging with up to 5, 7, 8, and 11 promoters, respectively. In contrast, promoter nodes typically have higher degrees than enhancers, particularly in the NHEK cell line, where some promoters are regulated by up to 30 enhancers, possibly indicating a more central role for promoters within the regulatory networks. Further examination of the degree distribution graphs reveals that the degree distribution in the IMR90 cell line is relatively “broad and short,” whereas it is “tall and thin” in the NHEK cell line. This suggests that the EPI network in IMR90 is simpler, with most nodes having fewer connections and more specific regulation, while the EPI network in NHEK is more complex, with more extensive regulatory relationships. To further understand the structure of the EPI network, we fitted the node degree distribution to common network distribution models, such as power-law, log-normal, and long-tail distributions. The fitting results showed that the p-values for all models were above 0.05, indicating that the degree distribution of the EPI network significantly differs from common protein interaction networks and may possess more complex and specific local structures. This phenomenon could be due to the highly specialized interaction patterns formed by enhancers and promoters for precise gene expression regulation.

**Figure 7:**
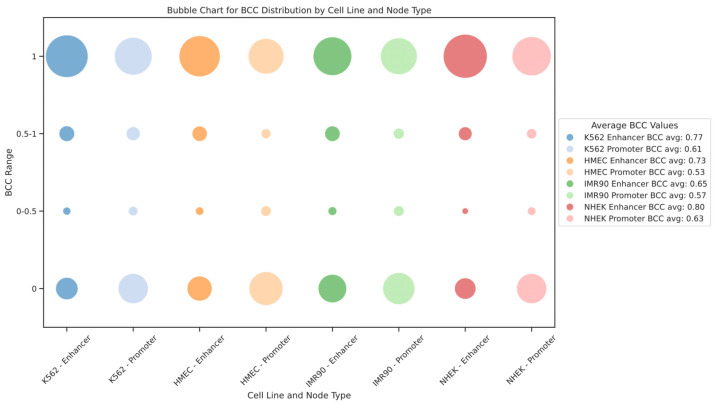
Distribution of bipartite clustering coefficients for enhancers and promoters across different cell lines. This figure illustrates the distribution of bipartite clustering coefficients (BCCs) within the EPI network across various cell lines, using a bubble chart to visually represent the data. The size and color of each bubble vary to indicate the BCC values for enhancer and promoter nodes, reflecting their tendency to form dense regulatory clusters or isolated interaction pairs. Higher BCC values suggest that enhancers and promoters are part of highly interconnected clusters, facilitating complex gene regulation. In contrast, lower BCC values indicate more isolated interactions, where nodes typically regulate fewer genes. This distribution highlights the complexity of regulatory networks and the role of spatial clustering in gene expression regulation.

We also modeled the EPI as a bipartite network. In this context, the bipartite clustering coefficient (BCC) quantifies the clustering between different types of nodes. BCC values range from 0 to 1, where BCC = 1 indicates that the nodes participate in a highly connected local network, and BCC = 0 indicates very low clustering between nodes. For the EPI network, BCC = 1 means that enhancers and promoters form a densely interconnected local structure, where multiple enhancers and promoters form a “cluster” through mutual connections. Such cluster formations reflect the synergy in gene regulatory networks, where multiple enhancers regulate gene expression through shared promoters or several promoters collaborate via a single enhancer. Conversely, BCC = 0 suggests that the enhancer and promoter form only an isolated interaction pair, without further regulatory connections. In such cases, the interactions between enhancers and promoters exhibit high specificity, as they tend to regulate only a single gene or a small group of genes rather than participating in complex regulatory networks. This implies that these enhancers and promoters may undertake more specific or independent regulatory functions within the gene regulatory network. The distribution of the bipartite clustering coefficient BCC for enhancer and promoter nodes in each cell line is shown in Figure 7. We found that the BCC values for enhancer nodes are generally higher than those for promoter nodes across different cell lines, indicating stronger clustering of enhancers within the gene regulatory network. Additionally, the distribution of BCC values shows extremes, with most nodes having BCC values of either 1 or 0, rarely in between, suggesting that the EPI network comprises both highly dense regulatory clusters and numerous isolated interaction pairs. This dual structure reveals the complexity and cell-type specificity of gene expression regulation within the network.

**Figure 8:**
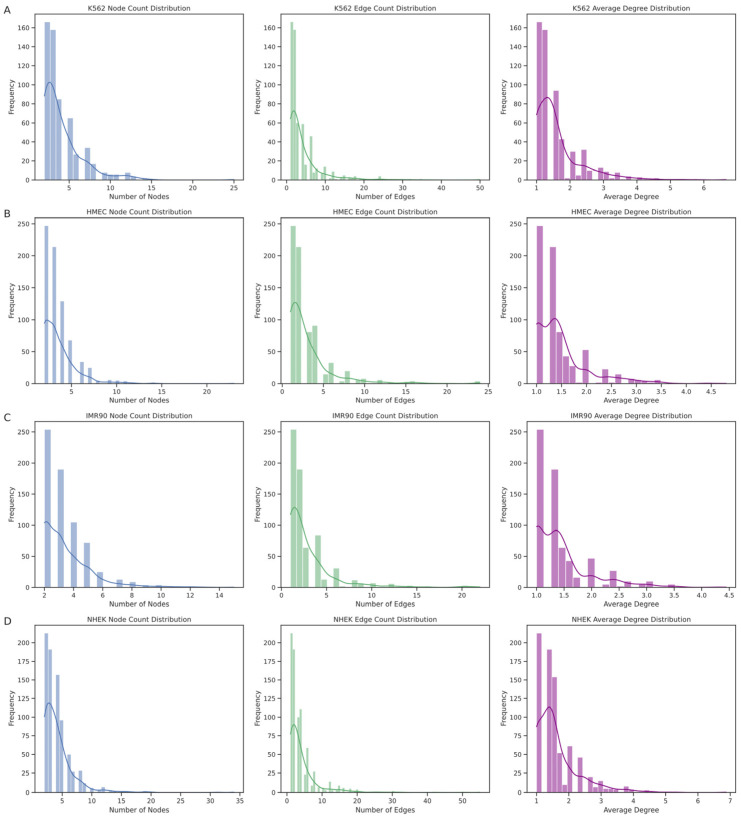
Structural distributions of connected subgraphs across different cell lines. This figure displays the structural characteristics of connected subgraphs within the enhancer–promoter interaction (EPI) networks for four distinct cell lines: (**A**) NHEK, (**B**) IMR90, (**C**) HMEC, and (**D**) K562. Panels from left to right represent the distributions of node counts, edge counts, and average node degrees within each subgraph. The node count distribution predominantly shows smaller values, indicating that most subgraphs consist of few nodes, thus implying limited connectivity. The edge count and average degree distributions further support this observation, reflecting sparse connections between regulatory elements. Interestingly, the K562 and NHEK cell lines exhibit some larger and denser subgraphs, suggesting more intricate regulatory clusters that might play significant roles in gene expression regulation. This variation suggests a diverse architectural landscape of enhancer–promoter interactions, which could underlie cell-type-specific regulatory complexities.

The EPI network consists of numerous discrete connected subgraphs, forming a broadly distributed archipelago network structure. By analyzing each connected subgraph’s node count, edge count, and average node degree (Figure 8), we further understood the unique enhancer–promoter interaction patterns within each cell line. The results show that the node count distribution is concentrated at smaller values, indicating that most enhancers and promoters in these networks interact with only a few other elements. Similarly, the distribution trends of edge counts and average node degrees align with the node count distribution, mostly falling within lower ranges, suggesting that most subgraphs have relatively sparse internal connections. However, we also observed a few larger subgraphs with higher distribution values, such as in the K562 cell line, where the largest subgraph had 25 nodes with an average node degree of 6.6667, and in the NHEK cell line, where the largest subgraph had 34 nodes with an average node degree of 6.8750, hinting at larger and denser regulatory clusters within the EPI network. The largest regulatory clusters in the HMEC and IMR90 EPI networks might not be as complex as those in K562 and NHEK. This structural diversity is likely crucial for the complex gene expression regulation mechanisms within cells.

Through these analyses, the EPI network in the IMR90 cell line exhibits high specificity due to the localized and limited interactions between enhancers and promoters. In contrast, the interactions between promoters and enhancers are more complex in the NHEK and K562 cell lines. These findings reveal unique regulatory patterns and structural characteristics of the EPI network across different cell lines, showing more complex regulatory dynamics and specific biological functions compared to common protein interaction networks. Although the EPI network exhibits certain archipelago characteristics and local clustering, its internal structural heterogeneity and complexity reveal the complexity and cell-type specificity of gene expression regulation.

## 4. Discussion

In the study of enhancer–promoter interaction (EPI) prediction, this paper introduces a novel approach based on graph neural networks, named GATv2EPI. Traditional EPI prediction methods typically consider simple one-to-one interactions between enhancers and promoters, which fail to capture the complexity of interactions across broader genomic regions. GATv2EPI addresses this limitation by constructing complex EPI network structures and leveraging GATv2 to utilize global topological information within the network, effectively capturing the intricate relationships between enhancers and promoters. Furthermore, in terms of feature selection, GATv2EPI distinguishes itself from most deep learning models for EPI prediction, which primarily use DNA sequence features of enhancers and promoters. Instead, GATv2EPI incorporates local epigenetic features (such as CTCF, DNase-seq, and histone modifications), along with spatial distance features between enhancers and promoters, providing richer information for EPI prediction. Regarding feature extraction regions, unlike DeepTACT, our method not only considers the features of the enhancers and promoters themselves but also integrates information from their flanking regions, offering a more comprehensive context that enhances the model’s generalization ability. In contrast to TransEPI, which extracts features within a 2.5 Mbp range, our approach requires only a 40 kb range, significantly reducing computational complexity and improving training efficiency. Additionally, GATv2EPI employs a novel dataset partitioning method based on graph connectivity, sampling all connected subgraphs for each chromosome.

This strategy effectively avoids the overestimation of model performance caused by the random partitioning used in methods like SIMCNN, EPI-Trans, TargetFinder, and ChINN. It also overcomes the limitations of chromosome-wise partitioning in TransEPI, where samples from the same chromosome are confined to a single dataset, ensuring biological consistency and independence in training, validation, and test sets, thereby enhancing the model’s reliability.

A limitation of our method is that, although we have reduced computational complexity by optimizing the range of feature extraction and using chromosome-based subgraph partitioning to save memory and shorten training time, GATv2EPI, being a graph neural network-based approach, still requires considerable computational resources when dealing with particularly large datasets. This can be a challenge in resource-limited research settings and may restrict the broader application of the model.

This study further underscores the critical role of epigenetic marks in regulating gene expression, particularly by enhancing the predictive model’s performance through the integration of local epigenetic information with the spatial relationships between enhancers and promoters. We systematically analyzed the structural characteristics of the EPI network and discovered that it functions as a highly localized archipelago network, with differences in EPI network structures among the various cell lines. K562 and NHEK exhibited higher bipartite clustering coefficients and larger, more densely connected subgraphs compared to HMEC and IMR90.

In our analysis of the EPI network, we believe that beyond the identified cell-line-specific enhancer–promoter interactions, future work should focus on extracting and identifying key regulatory subnetworks that may comprise multiple enhancers and promoters whose synergistic effects are crucial for the expression of specific genes. Moreover, mining EPIs related to diseases will provide important insights into disease mechanisms. By comparing the EPI network differences between healthy and diseased cell lines, specific enhancer–promoter interactions can be identified, which may play pivotal roles in the onset and progression of diseases. For instance, the fewer shared EPIs in the K562 cell line compared to normal cell lines might indicate specific regulatory mechanisms associated with cancer. Future research could systematically identify and validate these disease-related EPIs in conjunction with clinical data, thus providing potential biomarkers for precision medicine and targeted therapy.

Regarding the future development of EPI prediction models, we currently rely primarily on bulk data for analysis. While this approach captures overall biological features to some extent, its resolution and accuracy are limited. Our future goal is to achieve more refined analyses, delving into single-cell resolution using single-cell RNA-seq data to enhance the accuracy and biological relevance of EPI predictions. However, the current lack of single-cell ChIP-seq data restricts research in this area. With technological advancements and the successful application of single-cell sequencing technologies, we anticipate integrating more single-cell data to achieve more comprehensive and in-depth EPI predictions. This will provide stronger support for understanding complex biological processes and significantly enhance the model’s practical utility and scientific value.

In downstream applications, EPI prediction models can be extended to drug development, disease mechanism research, and personalized medicine. For example, based on EPI prediction results, researchers can identify potential therapeutic targets, accelerating new drug development. Furthermore, the model can be employed to study the changes in gene regulatory networks under specific disease states, aiding in the identification of disease-related biomarkers and providing critical guidance for diagnosis and treatment.

## Figures and Tables

**Table 1:** Summary of datasets after data preprocessing.

Cell Lines	Positive_Sample	Negative_Sample
HMEC	2153	2246
IMR90	1808	1939
NHEK	3092	3274
K562	2350	2381

**Table 2:** Comparison of training times for TransEPI and GATv2EPI across different cell types.

Cell Type	TransEPI Training Time	GATv2EPI Training Time
Normal	1001.41 s	47.18 s
Cancer	396.57 s	44.24 s

**Table 3:** Shared enhancer–promoter interactions across four cell lines.

Enhancer	Gene
EH37E0686948	*ENSG00000114107*
EH37E0126485	*ENSG00000153029*
EH37E0604662	*ENSG00000236352*
EH37E0126484	*ENSG00000153029*
EH37E0453734	*ENSG00000177426*
EH37E0207056	*ENSG00000133805*
EH37E0453734	*ENSG00000266578*
EH37E0207057	*ENSG00000133805*
EH37E0706618	*ENSG00000214145*

## Data Availability

The source code and datasets for the GATv2EPI project are hosted on GitHub and can be accessed at https://github.com/xjzhao123/GATv2EPI, accessed on 30 October 2024.
